# Task Sharing in Hearing Care: Putting Principles Into Practice to Advance Access to Hearing Care

**DOI:** 10.1093/geroni/igaa057.2932

**Published:** 2020-12-16

**Authors:** Nicole Marrone, Aileen Wong, Maia Ingram, Rosie Piper, Scott Carvajal, Sonia Colina

**Affiliations:** 1 University of Arizona, Tucson, Arizona, United States; 2 Arizona Prevention Research Center, Tucson, Arizona, United States; 3 Mariposa Community Health Center, Nogales, Arizona, United States

## Abstract

Task sharing, through models such as community health workers (CHWs), is considered an efficacious and cost-effective approach to extending access, addressing disparities, and building capacity. Increasingly, task sharing is recognized as a promising approach within sensory health. This session will share results from an NIH-funded trial of a first-in-kind CHW-delivered intervention along the U.S.-Mexico border. Trained CHWs provided a 5-week group aural rehabilitation program that included education and counseling on age-related hearing loss. A total of 136 Spanish-speaking older adults with hearing loss were randomized. Those in the immediate treatment group reported significantly greater use of communication strategies post-intervention, which was maintained over 1 year. Participants were more likely to report taking action on their hearing at 6 months (OR:1.56, p=0.001) and 1 year (OR:1.82, p=0.001). Building upon lessons learned, including post-intervention focus groups, the presentation will share guiding principles on the application of task sharing to support sensory health.

